# Evaluation of Antioxidative Activity of *Agrimonia pilosa-Ledeb* Leaves on Non-lipid Oxidative Damage

**DOI:** 10.5487/TR.2009.25.4.243

**Published:** 2009-12-30

**Authors:** Dae-Sik Hah, Chung-Hui Kim, Euikyung Kim, Jong-Shu Kim

**Affiliations:** 112 Gyeongnam Livestock Promotion Institute Middle-branch, Changwon, 541-703 Korea; 212 Department of Animal Science and Biotechnology, Jinju National University, Jinju, 660-758 Korea; 312 College of Veterinary Medicine, Gyeongsang National University (Institute of Animal Science), Jinju, 660-701 Korea

**Keywords:** Antioxidative activity, *Agrimonia pilosa leaves*, 2’-Deoxyguanosine (2’-dG), Non-lipid oxidative model systems

## Abstract

Present study was conducted to evaluate the antioxidative activity of the *Agrimonia pilosa-Ledeb* leaves on non-lipid oxidative damage. The antioxidative activity of methanolic (MeOH) extract of the *Agrimonia pilosa-Ledeb* leaves on non-lipid oxidation, including liposome oxidation, deoxyribose oxidation, protein oxidation, chelating activity against metal ions, scavenging activity against hydrogen peroxide, scavenging activity against hydroxyl radical and 2’-deoxyguanosine (2’-dG) oxidation were investigated. The MeOH extract of the *Agrimonia pilosa-Ledeb* leaves exhibited high antioxidative activity in the liposome model system. Deoxyribose peroxidation was inhibited by the MeOH extract of the *Agrimonia pilosa-Ledeb* leaves and MeOH extract of the *Agrimonia pilosa-Ledeb* leaves provided remarkable protection against damage to deoxyribose. Protective effect of MeOH extracts of the *Agrimonia pilosa-Ledeb* leaves on protein damage was observed at 600 µg level (82.05%). The MeOH extracts of the *Agrimonia pilosa-Ledeb* leaves at 300 µg revealed metal binding ability (32.64%) for hydrogen peroxide. Furthermore, the oxidation of 2’-deoxyguanosine (2’-dG) to 8-hydroxy-2-deoxyguanosine (8-OH-2’dG) was inhibited by MeOH extracts of the *Agrimonia pilosa-Ledeb* leaves and scavenging activity for hydroxyl radical exhibited a remarkable effect. From the results in the present study on biological model systems, we concluded that MeOH extract of the *Agrimonia pilosa-Ledeb* leaves was effective in the protection of non-lipids against various oxidative model systems.

## INTRODUCTION

Free radicals such as reactive oxygen species (ROS) are formed during a variety biological and cellular functions in living organisms. All cells having aerobic respiration, using oxygen as a nutrient, are continuously exposed to a number of exogenous and endogenous ROS or radicals that cause lipid, protein and DNA damage (Salles *et al.*, 1999; Droge, 2002). The endogenous source of ROS are formed from the mechanism of oxidative phosphorylation in which oxygen is reduced to water through a four step addition of electrons. The exogenous source of ROS are also produced by exposure to various environmental factors, for instance, UV light, ionizing radiations or tobacco (Hanato, 1995; Lander, 1997; Freidovich, 1999; Salles *et al.*, 1999; MeCord, 2000; Fang *et al.*, 2002). Antioxidant defenses have evolved to protect biological systems against reactive oxygen species, and a sophisticated, co-operative array of antioxidant defense mechanisms is found in biological systems. Antioxidant defenses system prevent generation and counteract the damaging effects of reactive species produced within the organism from molecular oxygen (Kimie *et al.*, 1988; Masaki *et al.*, 1995; Nobuyki *et al.*, 1996; Benzie, 2000). But an imbalance between the production of various reactive species and the ability of the organisms natural protective mechanism to cope with these reactive compounds was occurred, this condition was called oxidative stress. Oxidative stress causes a net stress on normal body functions and may result in many specific diseases. It also appears to contribute to the general decline in optimum body functions commonly known as ageing (Sohal and Weindruch, 1996; Wen *et al.*, 1999; Yun *et al.*, 2002; Sala *et al.*, 2003). Oxidative stress meditated many chronic fatigue disease such as Alzheimer's disease, autoimmune, diabetes, and cancer *et al.* (Floyd, 1990; Halliwell, 1991; Anne and Andrew, 2000; Selvendiran *et al.*, 2003; Valko *et al.*, 2007). From ancient, traditional medicinal plants have been known to posse antioxidative activity (Pratt and Watts., 1964; Fukuzawa and Takaishi, 1990; Inatain *et al.*, 1996). For several years, there are current interested in the development of therapeutic and chemopreventive antioxidative agents which have non-cytotoxicity. Many researchers suggest that antioxidants, in particular plants diet-derived antioxidants, might have health benefits as prophylactic agents (Aruoma *et al.*, 1999).

The *Agrimonia pilosa-Ledeb. Rosaceae* is traditional medicinal plant, which have already been known to have diverse properties such as antioxidative activity including antibacterial, anti-inflammatory, antipyretic, haemostatic, vasoconstrictor, analgesic and anti-tumor (Sala *et al.*, 2003).

Although, the protective or remedy mechanism of *Agrimonia pilosa*-Ledeb have not been fully elucidated, It has been commonly used in Korea and other Asian countries such as China, India , Japan as a natural flavoring agent for prevent, or treatment of various chronic-fatigue syndrome diseases (Su *et al.*, 1984; Isao *et al.*, 1988; Pei *et al.*, 1990; Cha *et al.*, 1997; Sala *et al.*, 2003). Therefore, considerable attention has been focused on quenching free radical oxidation from the natural medicinal plants and synthetic phenolic antioxidants such as butylated hydroxytoluene (BHT), butylated hydroxyanisole (BHA) are commonly added to foods to inhibit free radical damage to lipid; however, BHA, BHT are suspected as possible carcinogens (Branen, 1975; Duh *et al.*, 1999). Therefore, It seems to be need to find out the nature anti-oxidants without side effect from natural medicinal plants. Also, where or not occurred oxidative stress on non-lipid and/or be able to protect oxidative damage against free radicals using *Agrimonia pilosa*-Ledeb are not under clean. Thus, the objectives of present study were conducted to evaluate the antioxidative activity of *Agrimonia pilosa-Ledeb* leaves methanolic extract on non-lipid oxidative damage.

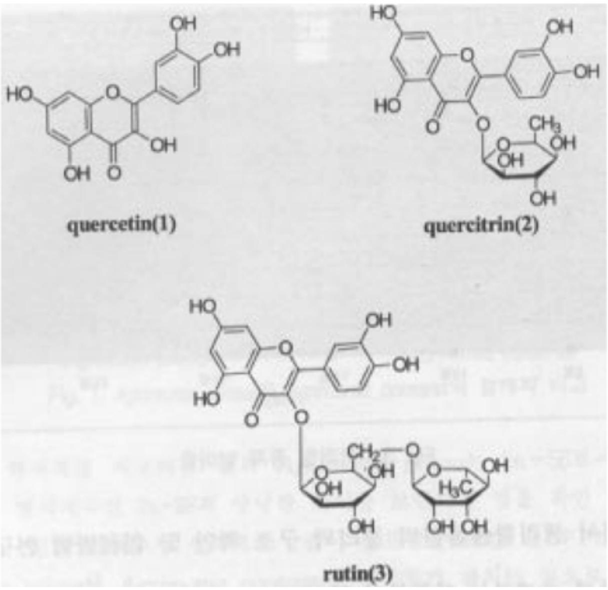

**Fig. 1.** Chemical structure of *Agrimonia pilosa* leaves.

## MATERIALS AND METHODS

***Plants materials and extracts preparation.*** The *Agrimonia pilosa* leaves were obtained from the Research Institute of Traditional Medicine Plants of Gyeongnam (Hamyang, Gyeongnam, Korea) and was identified by the head of Research Institute. Voucher specimens were deposited in the Research Institute and Its chemical structure shown in Fig. 1.

*Agrimonia pilosa* leaves were air dried under shade and cut into small pieces and stored at 4°C until use.

*Agrimonia pilosa* leaves 300 g were extracted with 80% 900 ml methanol in a shaking incubator at 80°C for 12 hr. The residue was re-extracted under the same condition 3 times. The extracts obtained were combined and filtered. The combined methanol specimens were evaporated to dryness using a vacuum rotary evaporator and weighted (98.88 g dry base) to determine the yield of soluble constituents. The extract obtained was subject to evaluate the antioxidative activity on non-lipid oxidative damage.

***Determination of the antioxidative activity on liposome peroxidation.*** 580 mg Lecithin (ICN, USA) containing 58 ml, 10 mM phosphate buffer (pH 7.4) was ultrasonicated in ultrasonic cleaner (Branson B-221 Smithkline Company, USA) for 2 hours. The sonicated solution (10 mg lecithin/ml), $Fecl_3$, ascorbic acid and MeOH extract (50~1200 μg) were mixed to produced a final concentration of 3.12 mg lecithin/ml, 125 μM $FeCl_3$, and 125 μM ascorbic acid. The mixture was incubated for 1 hr at 37°C by the thiobarbituric acid (TBA) method (Tamura and Shibamoto, 1991). The absorbance of the mixture was read at 532 nm against a blank, which contained all reagents except lecithin. The extinction coefficient of TBA-malonaldehyde product of 1.56 × $10^5$ $M^{-1}$ $cm^{-1}$ was used to convert absorbance values into concentrations of secondary reaction products.

***Determination of the antioxidative activity on protein carbonyl.*** The reaction mixture (1.2 ml), containing MeOH mixture (50~1200 μg), phosphate buffer (20 mM, pH 7.4), bovine serum albumin (20 mg/ml), $FeCl_3$ (100 μM), $H_2O_2$ (2.0 mM), ascorbic acid (200 μM), was incubated for 1 hr at 37°C, and 1 ml 20 mM in 2 M HCl was added to the reaction mixture and centrifuged at 3000 g for 10 min. The protein was washed three times with 2 ml ethanol-ethyl acetate (1:1) and dissolved in 2 ml 6 M guanidine-HCl (pH 6.5). The absorbance of the sample was read at 370 nm (Lenz *et al.*, 1989). All analyses were run in three replicates and averaged.

***Measurement of the effect on oxidation of deoxyribose.*** The reaction mixture (3.5 ml), which contained MeOH mixture (50~1200 μg), deoxyribose (6 mM), $H_2O_2$ (3 mM), $KH_2PO_4$-$K_2HPO_4$ buffer (20 mM, pH 7.4), $FeCl_3$ (400 μM), ethyenediaminetetraacetic acid (EDTA; 400 μM) and ascorbic acid (400 μM) was incubated at 37°C for 1 hr. The extent of deoxyribose degradation was tested by the TBA method. 1 ml 1% TBA and 1 ml 2.8% trichloroacetic acid (TCA) were added to the mixture, which was then heated in a water bath at 90°C for 20 min. The absorbance of the mixture was read spectrophotometerically at 532 nm (Halliwell *et al.*, 1987). All analyses were run in three replicates and averaged.

***Measurement of chelating activity on metal ions.*** The chelating activity of MeOH extract on $Fe^{2+}$ was measured according to the Carter method (1971). The MeOH extract (0.2 ml, 50~1200 μg), was incubated with 0.05 ml $Fecl_2$ $4H_2O$ (2.0 mM). the reaction mixture was initiated by the addition of 0.2 ml ferrozine (5.0 mM), and finally quantified to 0.8 ml with methanol. After the mixture had reached equilibrium (10 min), the absorbance at 562 nm was read. EDTA served as the positive control, and an untreated sample served as the negative control. All analyses were run in three replicates and averaged.

***Determination of the antioxidative activity on hydrogen peroxides.*** Antioxidative activity of MeOH extract to scavenge hydrogen peroxides was measured according to the Ruch *et al.* (1989). The MeOH extract (50~1200 μg) was added to hydrogen peroxide solution (0.6 ml, 2 mM) preparing in phosphate buffer saline (PBS, pH 7.4, at 20°C). MeOH extract was decolorized with cartridge (Sep-pack C18, Waters USA). Absorbance of hydrogen peroxide at 230 nm was determined 10 min later in a spectrophotometer against a blank solution containing MeOH extract in PBS without hydrogen peroxjde. Hydrogen peroxide concentration was determined spectrophotometrically at 230 nm using a molar extraction coefficient for hydrogen peroxide of 81 $M^{-1}$ $cm^{-1}$.

***Assay of the antioxidative activity on hydroxyl radicals.*** Scavenging effect of MeOH extract on hydroxyl radicals was assayed by the Shi *et al.* (1991) method. Briefly, The reaction solution containing 3 ml sodium phosphate buffer (0.15 mM, $KH_2PO_4$-$K_2HPO_4$, pH 7.4) was initiated by the addition of 100 μM vitamin C, 100 μM $CuSO_4$, 12 μM cytochrosome C. After initiation, the MeOH extract (50~1200 μg) was added to reaction mixture and incubated at 25°C for 90 min. The absorbance of the color changed in cytochrome C was read at 550 nm. Thiourea served as the positive control, Inhibition rate (%) of hydroxyl radical was calculated as following formula.

$$\text{Inhibition rate}\,(\%) = \frac{T - T2}{T - T1} \times 100$$

T: The transmittance of OH radical generation system,
T1: The transmittance of control system
T2: The transmittance of test sample system

***Assay of the antioxidative effect on 2'-deoxyuanosine oxidation.*** This assay was determined according to the Kasai and Nishimura method (1984). The reaction mixture (1.4 ml), containing MeOH mixture (50~1200 μg), 2'-dG (0.5 mM), $KH_2PO_4$-$K_2HPO_4$ buffer (0.1 M, pH 7.4), was initiated by the Fenton reaction model system, which contained $H_2O_2$ (50 mM), $FeCl_3$ $6H_2O_2$ (1.3 nM), EDTA (6.5 mM) and ascorbic acid (15 mM). All the mixture was incubated at 37°C for 30 min, and incubation was terminated by placing the mixture in an ice-bath, and then filtering through a 0.45 μm filter before use. The filtrate was analyzed by HPLC (Water, USA), using the Water E2241 F (103 mm × 1 mm, 0.5 μm) and UV detector (measured at 254 nm). The column was equilibrated with 50 mM $KH_2PO_4$ (pH 4.61)-methanol (93.5:6.5, v/v) at a flow rate of 0.5 ml/min. 2'-dG and 8-OH-2'dG were identified by comparison of their retention times with those of known standards and determined by peak areas from the chromatograms. All analyses were run in three replicates and averaged.

***Statistical analysis.*** The results are expressed as mean ± standard deviation (S.D). Differences between groups were assessed by one-way analysis of variance using the SAS software package for Windows. If in a one-way analysis of variance test a significant F-value of $p < 0.05$ was obtained, a Dunnett's multiple comparison test between the treated and control group was conducted. Differences between two groups were statistically evaluated by a t-test.

## RESULTS

***Determination of the antioxidative activity on liposome oxidation.*** Phospholipids which named as derivatives of phosphatidic acid such as phosphatidylcholine (lecithin) are the main components of cell membrane. To evaluate antioxidative activity of MeOH extract in a liposome model system, The lecithin, prepare as a liposome, was used. Antioxidative effect of MeOH extract against the inhibitory activity of lipid peroxidation in cell membranes were assessed by the amount of malondialdehyde (MDA) produced. MeOH extracts in the range of 100~600 μg showed 16.28~30.12% inhibition of peroxidation. The incidence of observed inhibition was dependent on amount up to 600 μg, but inhibition was decreased (29.14%) at 1200 μg. As results to compare the MeOH extract with butylated hydroxyanisol (BHA), butylated hydroxytoluene (BHT), and tocopherol (TCP) currently used as commercial antioxidants, BHA, BHT, and TCP, which at 600 μg showed 17.85%, 52.68% and 11.27% inhibition of peroxidation, respectively. Obviously, BHT at 600 μg showed a higher antioxidative activity than did 600 μg MeOH extract, However, BHA and TCP were lower than that of MeOH extract (Fig. 1).

***Determination of the antioxidative activity on protein carbonyl.*** The effect of MeOH extract on protein carbonyl formation in albumin, induced by $FeCl_3$, $H_2O_2$,

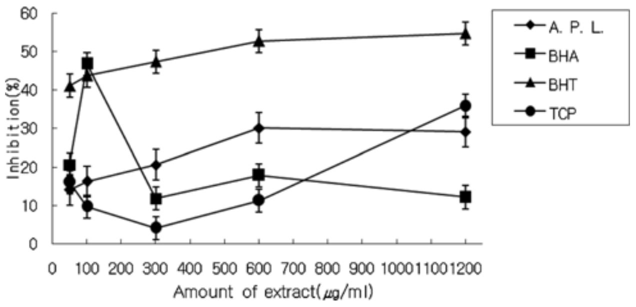

**Fig. 1.** Antioxidative effect of methanolic extract at different concentrations of the *Agrimonia Pilosa* leaves on liposome model system. A.P.L.: *Agrimonia Pilosa* leaf, BHT: butylated hydroxytoluene, BHA: butylated hydroxyanisole, TCP: tocopherol.

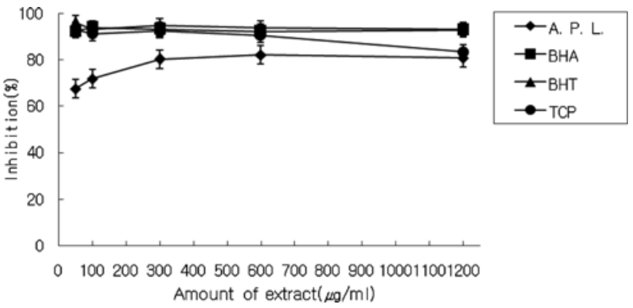

**Fig. 2.** Antioxidative effect of methanolic extract at different concentrations of the *Agrimonia Pilosa* leaf on protein oxidative damage. A.P.L.: *Agrimonia Pilosa* leaf, BHT: butylated hydroxytoluene, BHA: butylated hydroxyanisole, TCP: tocopherol.

and ascorbic acid was showed in Fig. 2. At 100~600 μg amounts of MeOH extract used, the inhibition of protein oxidation increased with increasing concentration of MeOH extract. As results to compare the MeOH extract with BHA, BHT, and TCP currently used as commercial antioxidants, BHA, BHT, and TCP, which at 100~1200 μg showed 92.92~94.25%, 92.92~94.75%, and 83.36~92.35% inhibition of protein carbonyl, respectively. Although, inhibition of protein carbonyl of MeOH extract was exhibited higher values (71.7~82.05%), its activity was lower than those of BHA, BHT, and TCP. But no significant difference ($p < 0.05$) in the values of protein carbonyl formation was found between the sample and comparative groups.

***Measurement of the effect on oxidation of deoxyribose.*** The antioxidative activity of MeOH extract on deoxyribose damage induced by $Fe^{3+}$/ $H_2O_2$, measured by the thiobarbituric acid method, was not amount-dependent. The extracts at the range of 100~1200 μg showed 63.32, 63.16, 67.19, and 66.37% inhi-

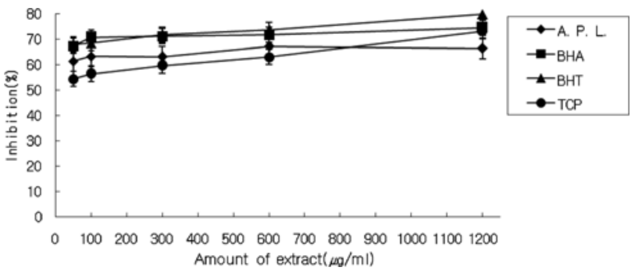

**Fig. 3.** Antioxidative effect of methanolic extract at different concentrations of the *Agrimonia Pilosa* leaf on deoxyribose oxidative damage. A.P.L.: *Agrimonia Pilosa* leaf, BHT: butylated hydroxytoluene, BHA: butylated hydroxyanisole, TCP: tocopherol.

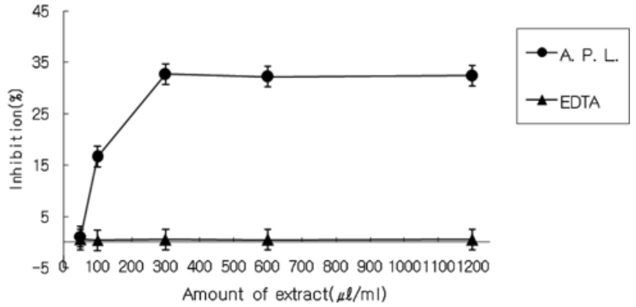

**Fig. 4.** Chelating effect of methanolic extract at different concentrations of the *Agrimonia Pilosa* leaf on ferrous ion. A.P.L.: *Agrimonia Pilosa* leaf, EDTA: Ethylenediaminetetraacetic acid.

bition of deoxyribose damage. Obviously, BHA and BHT at 100~1200 µg showed a higher inhibition of deoxyribose damage than did MeOH extract, but TCP was lower than that of MeOH extract except at 1200 µg (Fig. 3).

***Measurement of chelating activity on metal ions.*** The chelating effect of MeOH extract on ferrous ions is plotted in Fig. 4. As expected, the chelating effect increased with increasing amount of MeOH extract. MeOH extract at 300 µg has the highest effect on $Fe2^+$ binding (32.64%), whereas at 1200 µg MeOH extract showed a 32.35% chelating effect and 100 µg revealed the most lower chelating effect (16.63%). Although this amount (100 µg) has more lower value than the other amounts, it is relatively higher when compared with that of EDTA (2%). It may be significant because it minimizes the concentration of metal in the Fenton reaction.

***Determination of the antioxidative activity on hydrogen peroxides.*** The scavenging effect of different concentration of MeOH extract (100, 300, 600, and 1200 µg) on hydrogen peroxide was 5.45%, 24.39%, 23.71%, and 24.23%, respectively. At all amounts of MeOH used, the inhibition of hydrogen peroxides was more lower than those of BHA, BHT, and TCP. Especially, in the presence of 100 µg MeOH extract, almost incomplete suppression of hydrogen peroxides occurred, Whereas protection of hydrogen peroxides was 24.23% when the concentration of MeOH extract was at 1200 µg, indicating that the greater concentration (1200 µg) of MeOH extract, the less protection on hydrogen peroxides (Fig. 5).

***Assay of the antioxidative activity on hydroxyl radical.*** The scavenging effect of different amount of MeOH extract (50, 100, 300, 600, and 1200 µg) on hydroxyl radical was 75%, 80%, 86%, 90%, and 92%, respectively. MeOH extract markedly scavenged the hydroxyl radical , and Its scavenging effect increased with increasing amounts of MeOH extract as dose-dependent manner. Its scavenging effect was higher than BHA, TCP. But BHT was similar to the observation of MeOH extract (Fig. 6).

***Assay of the antioxidative effects on 2'-deoxyuanosine oxidation.*** The protective effect of MeOH extract on 8-OH-2'-dG formation exhibited 55~59.7% at range of 100~1200 µg. The incidence of 8-OH-2'-dG formation was decreased with increasing amounts of MeOH extract. When compared with the BHA, BHT, and TCP, MeOH extract posses less an inhibitory effect on the oxidation of 2'-dG (Fig. 7). The result of Fig. 7 was supported by Fig. 8 as showing with decreasing 8-OH-2'-dG formation than 2'-dG formation. The formation of 8-OH-2'-dG in the presence of $Fe^{3+}/EDTA/H_2O_2$ was analyzed by HPLC (water, USA). The retention time for 2'-dG and 8-OH-2'-dG were 4.517 and 5.358

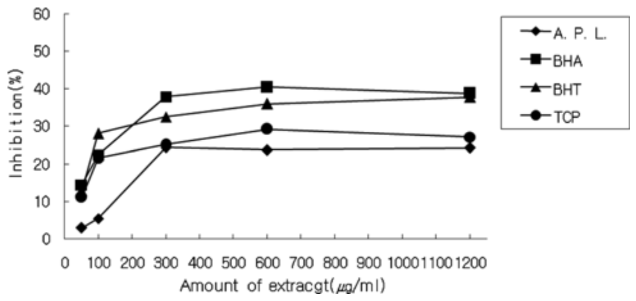

**Fig. 5.** Scavening effect of methanolic extract at different concentrations of the *Agrimonia Pilosa* leaf on hydrogen peroxide. APL: Agrimonia polisa leaves, BHA: Butylated hydroxyanisol. BHT: Butylated hydroxytoluene, TCP: Tocopherol.

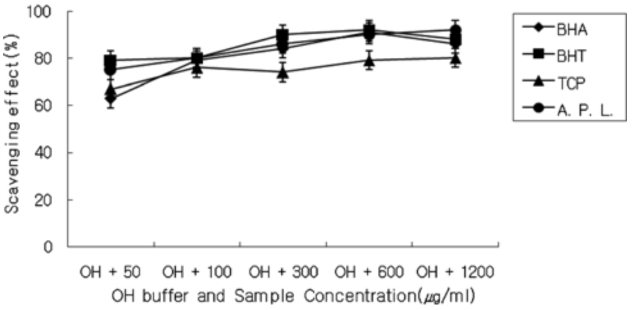

**Fig. 6.** Scavenging effect of methanolic extracts of *Agrimonia Pilosa* leaf on hydroxyl radical. A.P.L.: *Agrimonia Pilosa* leaf, BHT: butylated hydroxytoluene, BHA: butylated hydroxyanisole, TCP: tocopherol. OH: Hydroxyl radical generation system (100 µM Vitamin C, 100 µM $CUSO_4$, 12 µM Cytochrome C).

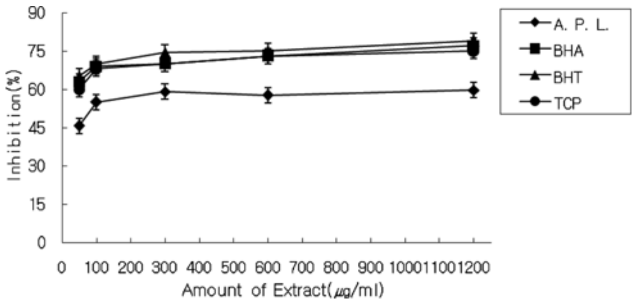

**Fig. 7.** Inhibitory effect of methanolic extracts of *Agrimonia Pilosa leaf* on 2'-deoxyguanosine (2'-dG) oxidative damage. A.P.L.: *Agrimonia Pilosa* leaf, BHT: butylated hydroxytoluene. BHA: butylated hydroxyanisole, TCP: tocopherol.

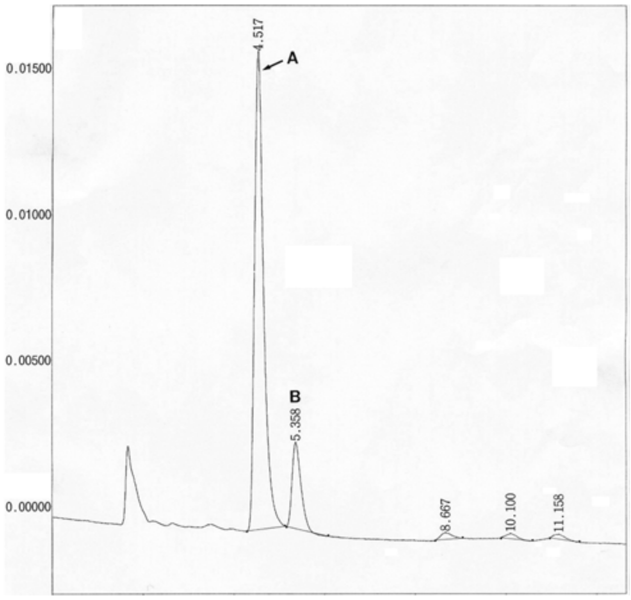

**Fig. 8.** HPLC chromatograms of 2'-deoxyguanosine oxidation. A: 2'-deoxyguanosine, B; 8-hydroxy-2'deoxyguanosine.

min, respectively (Fig. 8).

## DISCUSSION

In general terms, an antioxidant is anything which can prevent or inhibit oxidation (Halliwell, 1991). This can be achieved by preventing the generation of ROS, or by inactivating ROS (Benzie, 2000; Eklund *et al*., 2005). The membrane phospholipid is susceptible to free radical attack and leads to lipid peroxidation, membrane disruption, and changes the structure and function of key cellular constituents, resulting in mutation, cell damage and death (Chen and Stevens, 1991; Strain and Benzie, 1999; Halliwell and Gutteridge, 1987). Numerous studies suggested that unsaturated lipid, proteins, and DNA are the components of the cell that are most sensitive to oxidative damage induced by ROS. (Hatano, 1995; Masaki *et al*., 1995; Benzie, 2000; Karioti *et al*., 2004). The assessment of oxidative stress status was approached through the measurement the degree of oxidative damage on lipids, proteins, and DNA (Ayres, 1949; Ozcelik *et al*., 2003; Sloane and William, 1997; Kano *et al*., 2005). Although, antioxidative effect of MeOH extract during linoleic acid oxidation, determined by the DPPH and thiocyanate methods, was greater than BHA and TOC in a previous our study and because antioxidatine effect of control methanol solvent showed previous our study, its effect did not show in this study (Hah *et al*., 2005, 2007), the antioxidative activity of MeOH extract during lecithine liposome oxidation, determined by the TBA method in present study, was inferior to commercial BHA, and BHT. This result are consistent with previous studies showing that antioxidative activity of natural antioxidants was showed a wide range of activities and has different activity depending on substrates used and various system (Imaida *et al*., 1983; Frankel *et al*., 1997; Duh *et al*., 1999). Lipid peroxidation was measured by indirectly measure the MAD formed after lipid oxidation and MDA has been recognized as a good biomarker for lipid oxidation. MDA is associated with lipid oxidation and very reactive. Moreover, it can act as a catalyst in the formation of N-nitrosamines in foods containing secondary amines and nitrite (Eriksson, 1987). We selected methanol as extract solvent according to our previous study (Hah *et al*., 2005, 2007). From our results Fig. 1, MeOH extract showed significantly ($p < 0.05$) lower MDA when compared with BHA, TCP. This data implies that MeOH extract may protect against damage to cell membrane since they reduce the level of lipid peroxidation.

As showing the Fig. 2, The inhibition of protein oxidation increased with increasing amounts up to 600 μg (82.05%) and then decreased with increasing amounts at 1200 μg (80.7%). It seems to be that the protection of protein carbonyl formation was not complete. This finding is consistent with observation of Antosiewicz *et al*. (1995) who showed that inhibition effect of indolinic acid and quinolinic aminooxylson on protein of mice microsome and lipid oxidation. But disagree with published Mung bean Hull methanolic extract antioxidative activity (Duh, 1999). This difference was estimated by presuming that the components of sample used was not same sample used in this study.

Halliwell *et al*. (1987) and Aruoma (1991) report that $FeCl_3$-EDTA and $H_2O_2$ mixed solution is incubated with deoxyribose in phosphate buffer (pH 7.4), the hydroxyl radical formed attach to the deoxyribose and result serious of reactions that result in the formation of MDA. Consequently, the ability to diminish the amount of color formation has been adapted as one measurement of antioxidative properties. When any hydroxyl radical scavenger added to the this reaction mixture, It would compete with deoxyribose for hydroxyl radical and its concentration is higher than that of deoxylribose, hydroxyl radical was separated from deoxyribose to scavenger, deoxyribose was protected from hydroxyl radical (Zhao and Jung, 1995). This finding was found in present study, therefore, MeOH extract may be related to the protecting deoxyribose damage against oxidative stress. If some amino acid residues are oxidized, it convert to carbonyl derivatives; consequently, it could be used as one measure of protein damage (Stadman and Shibamoto, 1992; Neuzil *et al.*, 1993).

Iron and copper irons are powerful promoters of free radical damage, causing formation of hydroxyl radicals and accelerating lipid peroxidation (Gutteridge and Stocks, 1981). Antioxidant may have a direct antioxidative effect by scavenging free radicals as well as binding metal ions to reduced their absorption. The molecular that can inhibit deoxyribose degradation are those that can chelate the iron ions from the deoxyribose and render them inactive or poorly active in a Fenton reaction (Smith *et al.*, 1992). As showed in Fig. 4. the chelating effect of MeOH extract on metal ions may relate to their inhibition of deoxyribose degradation.

Superoxide can form hydrogen peroxide and this can, in turn, form the highly reactive hydroxyl radical. Removal of superoxide, therefore, is a key antioxidant defense mechanism (Fridovich, 1974). Hydrogen peroxide is one of the active oxygen specie capable of injuring DNA (Imlay and Linn, 1988). Indeed, $H_2O_2$ has been implicated in the induction of the nuclear transcription factor NFkB in Jurket cells (Ginn and Whisler, 1998; Schreck *et al.*, 1991). High concentration of $H_2O_2$ induced necrosis to cell while low concentration induced apoptosis (Lennon *et al.*, 1991). Hydrogen peroxide also induces DNA damage through Fenton reaction *in vivio* and *in vitro* (Aruoma *et al.*, 1999; Imlay and Linn, 1988). Hence, the ability of MeOH extract to scavenge hydrogen peroxide may contribute to the inhibitory effect on oxidative system.

The scavenge effect of MeOH extract on hydroxyl radical was increased with increasing amount. The inhibitory effect of antioxidants on the oxidative damage of non-lipids was due to the scavenging of hydroxyl radical, it is essential to evaluate whether antioxidants is able to scavenge hydroxyl radical or not. The results of Fig. 6. indicated that the marked inhibitory effect of MeOH extract on the oxidative damage of non-lipids may be concerned with its higher scavenging of hydroxyl radical.

The formation of 8-OH-2'-dG was induced by various environmental factors, such as ionizing radiation, UV light or Fenton reaction, indicating that the formation of 8-OH-2'-dG occurred through the reaction of DNA with hydroxyl radicals or singlet oxygen (Ames and Gold, 1991; Floyd, 1990). Kasai *et al.* (1986) mentioned that generation of oxygen radicals *in vivio* is thought to be relevant to carcinogenesis, and 8-OH-2'-dG formation in DNA may be related to tumor-genesis. As shown in Fig. 7. Although, the inhibitory effect on 8-OH-2'-dG oxidation damage was seems to be different from 55~59.7% depending amounts, We suggested that MeOH extract was expected for strong antioxidant to protect free radical inducing DNA damage.

In conclusion, the present results on biological model system showed that *Agrimonia pilosa* leaves was effective in the protection of lipids and non-lipids against various oxidative stress model system. This might be serving as a commercial, useful antioxidant in food processing and might be a potential adjutant antioxidant in pharmaceutical preparations or in organ preservation fluids where oxidative stability is desired. One of the major factors determining the antioxidative activity of *Agrimonia pilosa leaves* against ROS induced cell damage is may be due to its components*, quercetin, quercitrin, rutin.* However, It is difficult to identify the specific contribution of each single component about these effects. Which components critical role are under further investigation.

## Abbreviations

MeOH: methanol
2’dG: 2’-deoxyguanosine
8-OH-2’dG: 8-hydroxy-2-deoxyguanosine
